# Improving the Clinical Interpretability of Inpatient Blood Glucose Monitoring Through a Standardized Documentation Chart: A Quality Improvement Study at a Tertiary Public Hospital in the Caribbean

**DOI:** 10.7759/cureus.114677

**Published:** 2026-08-17

**Authors:** Christa Lyons

**Affiliations:** 1 Internal Medicine, University of the West Indies, Kingston, JAM

**Keywords:** blood glucose monitoring, clinical audit, diabetes mellitus, inpatient diabetes, quality improvement

## Abstract

Introduction

Accurate inpatient blood glucose documentation is essential for safe diabetes management, yet paper-based monitoring systems often record glucose values at clock time rather than during physiologically relevant monitoring periods. This quality improvement study evaluated whether implementing a standardized, timing-based diabetes monitoring chart improved the completeness and clinical interpretability of inpatient blood glucose documentation at a tertiary public hospital in the Caribbean.

Materials and methods

A two-cycle clinical audit was conducted over six months. Twenty-five adult medical inpatients with type 2 diabetes mellitus were evaluated before and after implementation of the intervention. The intervention consisted of a redesigned paper-based diabetes monitoring chart implemented hospital-wide, which recorded blood glucose measurements during pre-breakfast, pre-lunch, pre-supper, and bedtime monitoring periods, with adjacent documentation of insulin administration and staff education. The primary outcome was the proportion of clinically interpretable diabetes monitoring charts. Secondary outcomes included documentation of meal-related blood glucose timing, insulin administration in close proximity to blood glucose measurements, and incomplete diabetes monitoring charts.

Results

A total of 50 patient records were reviewed. Clinically interpretable documentation increased from 10/25 (40%) at baseline to 23/25 (92%, p < 0.001) following implementation. Documentation of meal-related blood glucose timing improved from 9/25 (36%) to 23/25 (92%, p < 0.001), while documentation of insulin administration adjacent to blood glucose measurements increased from 14/25 (56%) to 24/25 (96%, p < 0.001). Overall compliance with predefined documentation standards improved from 11/25 (44%) to 23/25 (92%, p < 0.001), and the number of incomplete diabetes monitoring charts decreased from 8/25 (32%) to 2/25 (8%, p = 0.074).

Conclusions

Implementation of a standardized timing-based diabetes monitoring chart was associated with marked improvements in the completeness and interpretability of inpatient blood glucose documentation. This simple, low-cost quality improvement intervention may enhance inpatient diabetes documentation in hospitals that continue to rely on paper-based medical records and could be adapted for use in other resource-limited healthcare settings.

## Introduction

Diabetes mellitus is one of the leading causes of morbidity and mortality worldwide and represents a major public health challenge [1]. Hospitalized patients with diabetes are particularly vulnerable to complications arising from acute illness, physiological stress, altered nutritional intake, and changes to their usual antihyperglycemic therapy [2]. Consequently, careful monitoring of inpatient blood glucose is essential for safe and effective clinical management.

Hyperglycemia during hospital admission has been associated with increased risks of infection, delayed wound healing, prolonged hospitalization, intensive care admission, and mortality [3]. Conversely, overly aggressive treatment may precipitate hypoglycemia, which is associated with neurological injury, cardiac arrhythmias, increased mortality, and longer hospital stays. For these reasons, maintaining appropriate inpatient glycemic control has become a key component of high-quality hospital care.

International organizations, including the American Diabetes Association (ADA), Joint British Diabetes Societies for Inpatient Care (JBDS-IP), and the National Institute for Health and Care Excellence (NICE), recommend that capillary blood glucose measurements be performed at meaningful time points, typically before meals and at bedtime in patients consuming a normal diet, or at regular intervals in patients who are fasting or receiving continuous enteral nutrition [4-6]. These recommendations recognize that interpretation of blood glucose values depends not only on the measured glucose concentration itself but also on the physiological context in which the measurement is obtained.

Despite these recommendations, documentation practices within many hospitals remain variable. In institutions that rely on paper-based medical records, blood glucose measurements are frequently documented by clock time alone, without indicating whether the readings were obtained before meals, after meals, or after insulin administration. Although this approach accurately records glucose values, it often limits their clinical interpretability. For example, a blood glucose concentration of 9.8 mmol/L may be interpreted differently depending on whether it was measured before a meal, after a meal, or following insulin administration. Without documentation of the timing of measurements relative to meals or medication, clinicians may find it difficult to accurately assess glycemic trends or make appropriate adjustments to insulin therapy.

This issue is particularly relevant in resource-limited healthcare settings, such as a tertiary public hospital in the Caribbean. Blood glucose measurements are documented by clock time on paper monitoring charts, requiring clinicians to consult medication administration records or nursing notes to determine the relationship between these measurements and meals and insulin administration. Informal feedback from physicians and nursing staff suggested that this documentation format complicated interpretation of glycemic control, increased workload, delayed insulin adjustment, and created opportunities for communication errors.

To address this issue, a standardized diabetes monitoring chart was developed in which blood glucose measurements were recorded pre-breakfast, pre-lunch, pre-supper, and at bedtime, with corresponding documentation of insulin administration. The intervention was accompanied by targeted education for nursing staff before hospital-wide implementation. This quality improvement study aimed to evaluate whether the intervention improved the completeness and clinical interpretability of inpatient blood glucose documentation among adult medical inpatients with diabetes mellitus.

## Materials and methods

Study design and setting

This quality improvement study was conducted using a two-cycle clinical audit at a tertiary public hospital in the Caribbean. The audit was conducted over a six-month period. The audit evaluated the quality of inpatient blood glucose documentation before and after the hospital-wide implementation of a standardized diabetes monitoring chart. The study followed the conventional audit cycle of baseline measurement, intervention, and re-audit to determine whether changes in documentation practice resulted in improved compliance with predefined documentation standards.

To minimize potential sources of bias and confounding, both audit cycles were conducted within the same hospital using identical eligibility criteria, audit standards, data collection methods, and outcome definitions. Documentation quality was assessed by the same investigator throughout the study using a standardized audit pro forma to ensure consistency in data collection. However, because the intervention included staff education in addition to the introduction of the redesigned monitoring chart, some of the observed improvements may have reflected increased awareness of documentation practices and the revised documentation format.

Audit standards

The audit standards were developed using recommendations from the ADA, JBDS-IP, institutional diabetes management protocols, and local expert consensus. Documentation was assessed against five predefined standards, including (1) blood glucose measurements should be documented according to physiologically relevant monitoring periods (pre-breakfast, pre-lunch, pre-supper, and at bedtime); (2) insulin administration should be documented adjacent to the corresponding blood glucose measurement; (3) diabetes monitoring charts should be completed without missing entries where monitoring was indicated; (4) documentation should allow clinicians to interpret glycemic control without reference to additional medical records; and (5) monitoring charts should be completed clearly and legibly. Overall compliance was defined as the fulfillment of all five predefined documentation standards in a patient’s diabetes monitoring chart.

Study population

Adult medical inpatients (≥18 years) with a documented diagnosis of type 2 diabetes mellitus who required routine capillary blood glucose monitoring during their admission were eligible for inclusion. Patients admitted for less than 24 hours, those receiving end-of-life care, patients managed exclusively in the intensive care unit, and patients without documented blood glucose monitoring were excluded.

Eligible patient records were identified from ward admission lists and corresponding diabetes monitoring charts during each audit cycle. Consecutive sampling was used, with the first 25 eligible patient records meeting the predefined inclusion criteria in each audit cycle included in the analysis. Consistent with the objectives of a quality improvement clinical audit and the Standards for Quality Improvement Reporting Excellence 2.0 guidelines, no formal a priori sample size calculation was performed [7]. Instead, a pragmatic sample of 25 patients per audit cycle was selected based on feasibility, the anticipated number of eligible admissions during each audit period, and established clinical audit methodology. The baseline and re-audit groups comprised separate patient cohorts; no patient was included in both audit cycles.

Baseline audit

During the initial audit cycle, diabetes monitoring charts were reviewed using a standardized audit pro forma. Data collected included patient age, sex, insulin therapy, length of admission, and completeness of documentation.

Intervention

The quality improvement intervention consisted of redesigning the existing paper-based diabetes monitoring chart. Rather than documenting blood glucose values by clock time alone, the revised chart recorded measurements by physiologically relevant monitoring periods: pre-breakfast, pre-lunch, pre-supper, and bedtime. Dedicated fields were also included to record insulin type, dose, and time of administration immediately adjacent to the corresponding blood glucose measurement. Additional space was provided for documenting episodes of hyperglycemia or hypoglycemia, omitted insulin doses, and other relevant clinical comments. Following approval by the hospital’s audit department, the revised chart was implemented hospital-wide. The unit head was responsible for sensitizing relevant staff to the revised documentation system. Staff were provided access to the approved document, instructed to review the changes, and allowed to clarify questions regarding its use. Staff subsequently signed a familiarization log confirming that they had reviewed the document and understood their responsibility to implement and adhere to the revised documentation requirements.

Re-audit

Following implementation of the revised monitoring chart and completion of staff education, a second audit cycle was performed using identical inclusion criteria, audit standards, and data collection methods. Documentation quality during the re-audit was assessed by the same investigator using the same standardized audit pro forma and predefined assessment criteria as those used during the baseline audit.

Outcome measures

The primary outcome was the proportion of diabetes monitoring charts considered clinically interpretable. Clinically interpretable documentation was defined as documentation that provided sufficient information to interpret the patient’s glycemic monitoring from the diabetes monitoring chart alone, without requiring reference to additional medical records. Secondary outcomes included documentation of meal-related blood glucose timing, insulin administration in close proximity to blood glucose measurements, and incomplete diabetes monitoring charts.

Statistical analysis

Data were analyzed using SPSS Statistics version 29.0 (IBM Corp. Released 2022. IBM SPSS Statistics for Windows, Version 29.0. Armonk, NY). Continuous variables were summarized using means and standard deviations (SD), while categorical variables were reported as frequencies and percentages. Comparisons between baseline and re-audit groups were performed using the independent-samples t-test for continuous variables and Pearson's chi-square test or Fisher's exact test for categorical variables, as appropriate. A two-sided p-value < 0.05 was considered statistically significant.

Ethical considerations

This project was undertaken as a quality improvement initiative to evaluate compliance with institutional standards for inpatient blood glucose documentation. As the project constituted a clinical audit of routine practice and involved anonymized data collected as part of standard clinical care, formal research ethics committee approval was not required according to institutional policy. The audit was approved by the hospital audit department prior to implementation of the revised diabetes monitoring chart. No patient-identifiable information was collected, and all data were analyzed anonymously.

## Results

Participant characteristics

A total of 50 patient records were reviewed during the study period, comprising 25 patients in the baseline audit and 25 patients in the re-audit following implementation of the standardized diabetes monitoring chart. Baseline demographic and clinical characteristics are summarized in Table 1.

**Table 1:** Demographic and clinical characteristics of patients in the baseline and re-audit groups Data are presented as mean ± SD, numbers (n), percentages (%), and p-values. Age is expressed in years and length of hospital stay in days. Comparisons between baseline and re-audit groups were performed using the independent samples t-test for continuous variables and Pearson's chi-square test or Fisher's exact test for categorical variables, as appropriate. Statistical significance was defined as p < 0.05. SD: standard deviation

Characteristic	Baseline audit (n = 25)	Re-audit (n = 25)	p-value
Age, years (mean ± SD)	63.4 ± 12.7	61.8 ± 13.5	0.67
Male sex	11 (44%)	12 (48%)	0.78
Female sex	14 (56%)	13 (52%)	0.78
Receiving insulin therapy	19 (76%)	20 (80%)	0.73
Length of hospital stay, days (mean ± SD)	7.8 ± 3.2	7.5 ± 2.9	0.73

There were no statistically significant differences in baseline demographic or clinical characteristics between the two audit cycles. The mean age was 63.4 ± 12.7 years at baseline and 61.8 ± 13.5 years at re-audit (p = 0.67). Most patients in both audit cycles received insulin therapy during admission, accounting for 19 (76%) patients in the baseline audit and 20 (80%) patients in the re-audit. Similarly, the mean duration of hospital stay did not differ significantly between the two groups (7.8 ± 3.2 vs. 7.5 ± 2.9 days, p = 0.73).

Documentation outcomes

Documentation outcomes before and after implementation of the standardized diabetes monitoring chart are presented in Table 2. The proportion of clinically interpretable diabetes monitoring charts increased from 10 patients (40%) during the baseline audit to 23 patients (92%) during the re-audit (p < 0.001). Documentation of blood glucose measurements during physiologically relevant monitoring periods also improved, increasing from 9 (36%) to 23 (92%) patients (p < 0.001).

**Table 2:** Documentation outcomes before and after implementation of the standardized diabetes monitoring chart Data are presented as numbers (n), percentages (%), and p-values. Absolute change represents the percentage-point difference between the baseline audit and re-audit. Comparisons between baseline and re-audit groups were performed using Pearson's chi-square test or Fisher's exact test, as appropriate. Statistical significance was defined as p < 0.05.

Outcome	Baseline audit (n = 25)	Re-audit (n = 25)	Absolute change (percentage points)	p-value
Clinically interpretable documentation	10 (40%)	23 (92%)	+52	<0.001
Meal-related glucose timing documented	9 (36%)	23 (92%)	+56	<0.001
Insulin documented adjacent to glucose measurement	14 (56%)	24 (96%)	+40	<0.001
Overall compliance with all five documentation standards	11 (44%)	23 (92%)	+48	<0.001
Incomplete documentation	8 (32%)	2 (8%)	−24	0.074

Documentation of insulin administration alongside the corresponding blood glucose measurement also increased from 14 (56%) patients before implementation to 24 (96%) patients following the introduction of the revised monitoring chart (p < 0.001). Overall compliance with the predefined documentation standards increased from 11 (44%) patients to 23 (92%) patients (p < 0.001), while incomplete diabetes monitoring charts decreased from eight (32%) patients to two (8%) patients (p = 0.074).

Overall, improvements were observed across all predefined documentation standards following implementation of the standardized diabetes monitoring chart. The largest absolute improvement was observed in documentation of meal-related blood glucose timing. In contrast, documentation of insulin administration adjacent to blood glucose measurements achieved the highest compliance during the re-audit. Incomplete documentation was markedly reduced following implementation of the intervention.

## Discussion

This quality improvement study demonstrated that implementing a standardized, timing-based diabetes monitoring chart was associated with improved completeness and clinical interpretability of inpatient blood glucose documentation. Following the introduction of the revised chart and staff education, compliance improved across all predefined audit standards, including documentation of meal-related blood glucose timing, insulin administration adjacent to blood glucose measurements, and overall adherence to the predefined documentation standards. Collectively, these findings demonstrate that a simple modification to an existing paper-based documentation system can substantially improve the quality and usability of inpatient blood glucose records without requiring substantial additional technological or financial resources.

A key improvement observed in this study was the documentation of blood glucose measurements according to physiologically relevant monitoring periods. Before the implementation of the intervention, glucose values were generally recorded based solely on clock time. Although the recorded values were numerically accurate, this format often required clinicians to consult medication charts or nursing notes to determine whether measurements were obtained before meals, after meals, or after insulin administration. The redesigned monitoring chart addressed this limitation by organizing documentation into pre-breakfast, pre-lunch, pre-supper, and bedtime monitoring periods, allowing blood glucose measurements and insulin administration to be reviewed together in a single document during ward rounds. These findings are consistent with previous studies demonstrating that standardized inpatient diabetes documentation improves the completeness and organization of clinical information. In particular, Kopanz et al. reported that implementing a standardized inpatient insulin chart significantly improved the structure and quality of diabetes documentation and reduced documentation errors, highlighting the importance of chart design in facilitating safe inpatient diabetes management [8].

Another notable finding was the improvement in documentation of insulin administration. Prior to implementation, insulin doses were frequently documented separately from blood glucose measurements, requiring clinicians to cross-reference multiple documents before making therapeutic decisions. Following the introduction of the revised monitoring chart, nearly all patient records documented insulin administration alongside the corresponding blood glucose measurement. This streamlined documentation process may facilitate more efficient review of blood glucose and insulin records during ward rounds and support communication among members of the multidisciplinary team. These findings are supported by prior work demonstrating that the design of inpatient insulin charts directly affects documentation quality. Kopanz et al. found that structural deficiencies in heterogeneous insulin charts were associated with frequent documentation and medication errors, highlighting the importance of standardized documentation tools in supporting safe inpatient diabetes care [9].

Other initiatives have focused on implementation of electronic prescribing systems, computerized decision support, specialist inpatient diabetes teams, or comprehensive educational programs [10-12]. These interventions have generally been associated with improvements in documentation quality, prescribing practices, or glycemic management but often require considerable technological infrastructure, financial investment, and organizational support. By comparison, the intervention evaluated in the present study consisted of redesigning an existing paper monitoring chart accompanied by brief staff education. No additional equipment, software, or specialist personnel was required. This simplicity is an important strength, particularly for hospitals operating in resource-limited settings where paper-based documentation remains the standard of care. Although electronic diabetes management systems have been shown to improve documentation quality, improvements in clinical outcomes have been less consistent, suggesting that optimizing documentation alone does not necessarily translate into better glycemic control. The findings of the present study demonstrate that substantial improvements in documentation quality can be achieved using a simple, low-cost intervention without the need for sophisticated digital systems.

The implications of this study extend beyond the Caribbean. Many healthcare systems in low- and middle-income countries continue to rely on handwritten clinical documentation, and implementing electronic prescribing systems may be infeasible due to financial or infrastructure constraints [13]. In these settings, relatively inexpensive quality improvement interventions that optimize existing documentation processes may yield meaningful gains in patient safety while requiring minimal additional resources. Furthermore, the principles underlying the redesigned monitoring chart could readily be adapted for institutions using electronic health records by incorporating physiologically relevant monitoring periods into digital documentation templates. Once introduced into routine clinical practice, the revised monitoring chart also requires minimal ongoing financial investment. Continued compliance could be supported through staff orientation, periodic educational updates, and regular clinical audits, enabling healthcare organizations to monitor adherence and identify opportunities to further refine documentation practices.

Although documentation quality improved substantially following implementation of the intervention, the present study assessed process measures rather than direct clinical outcomes. Accurate documentation is an important prerequisite for safe inpatient diabetes management, but improved documentation alone does not necessarily translate into improved patient outcomes. Future studies should evaluate whether improvements in documentation are sustained over longer follow-up periods and whether enhanced documentation is associated with reductions in hypoglycemic events, insulin prescribing errors, episodes of severe hyperglycemia, length of hospital stay, or inpatient mortality. Assessment of staff satisfaction, usability of the redesigned monitoring chart, and long-term sustainability would also provide valuable information regarding successful implementation and broader applicability.

This study has several limitations. First, it was conducted at a single tertiary public hospital. This may limit the generalisability of the findings to other healthcare settings with different patient populations, staffing structures, or documentation systems. Second, the study evaluated documentation quality rather than clinical outcomes. Although improved documentation is expected to facilitate safer and more effective diabetes management, the study was not designed to determine whether the intervention reduced medication errors, hypoglycemic episodes, or hospital length of stay. Third, because the intervention was accompanied by staff education, some of the observed improvements may reflect increased awareness of documentation practices rather than the redesigned monitoring chart alone.

Nevertheless, educational reinforcement is an integral component of many successful quality improvement initiatives and is routinely implemented in clinical practice. Fourth, the study included a relatively modest sample size of 50 patient records and used a pre/post design without a concurrent comparison group. While this was appropriate for a local clinical audit, larger multicenter studies with controlled designs would provide greater statistical precision and improve the external validity of the findings. Finally, documentation quality was assessed by a single investigator, which may have introduced observer bias, and staff awareness of the audit may have temporarily influenced documentation practices through a potential Hawthorne effect. However, predefined audit criteria and a standardized audit pro forma were used throughout both audit cycles to promote consistency.

## Conclusions

Implementation of a standardized timing-based diabetes monitoring chart was associated with improvements in the completeness and clinical interpretability of inpatient blood glucose documentation. Organizing blood glucose measurements by physiologically relevant monitoring periods and documenting insulin administration alongside corresponding glucose values substantially improved compliance with predefined documentation standards.

This quality improvement initiative suggests that simple, low-cost modifications to paper-based documentation systems can meaningfully enhance the quality of inpatient diabetes documentation without requiring expensive technological infrastructure. Similar interventions may be particularly valuable in hospitals that continue to rely on paper medical records and should be considered as part of broader strategies to improve inpatient diabetes care.

## References

[1] Hossain MJ, Al-Mamun M, Islam MR (2024). Diabetes mellitus, the fastest growing global public health concern: early detection should be focused. Health Sci Rep.

[2] Marín-Peñalver JJ, Martín-Timón I, Del Cañizo-Gómez FJ (2016). Management of hospitalized type 2 diabetes mellitus patients. J Transl Int Med.

[3] Farrugia Y, Mangion J, Fava MC, Vella C, Gruppetta M (2022). Inpatient hyperglycaemia, and impact on morbidity, mortality and re-hospitalisation rates. Clin Med (Lond).

[4] (2026). Diabetes testing and monitoring. https://diabetes.org/living-with-diabetes/treatment-care/checking-your-blood-sugar.

[5] (2026). Joint British Diabetes Societies (JBDS) for inpatient care group. https://abcd.care/jbds-ip.

[6] (2026). Type 2 diabetes in adults: management. https://www.nice.org.uk/guidance/ng28/chapter/Blood-glucose-management.

[7] Ogrinc G, Davies L, Goodman D, Batalden P, Davidoff F, Stevens D (2016). SQUIRE 2.0 (Standards for QUality Improvement Reporting Excellence): revised publication guidelines from a detailed consensus process. BMJ Qual Saf.

[8] Kopanz J, Sendlhofer G, Lichtenegger K (2021). Evaluation of an implemented new insulin chart to improve quality and safety of diabetes care in a large university hospital: a follow-up study. BMJ Open.

[9] Kopanz J, Lichtenegger KM, Sendlhofer G (2021). Limited documentation and treatment quality of glycemic inpatient care in relation to structural deficits of heterogeneous insulin charts at a large university hospital. J Patient Saf.

[10] Konnyu KJ, Yogasingam S, Lépine J (2023). Quality improvement strategies for diabetes care: effects on outcomes for adults living with diabetes. Cochrane Database Syst Rev.

[11] Flanagan D, Avari P, Choudhary P, Lumb A, Misra S, Rayman G, Dhatariya K (2023). Using technology to improve diabetes care in hospital: the challenge and the opportunity. J Diabetes Sci Technol.

[12] Sheikh A, Coleman J, Chuter A (2022). Electronic prescribing systems in hospitals to improve medication safety: a multimethods research programme. Programme Grants Appl Res.

[13] Fatajo E, Sa'ad T (2026). Exploring the challenges of paper-based documentation in clinical and administrative departments at Edward Francis Small Teaching Hospital, The Gambia. Ayden Int J Bank Finance Technol.

